# Alpha-Lipoic Acid in Depression and Cognitive Disorders: A Systematic Review

**DOI:** 10.1192/j.eurpsy.2026.11386

**Published:** 2026-07-31

**Authors:** M. F. Zeilstra, M. Arts, K. M. Zeilstra-Kalt, L. de Jonge

**Affiliations:** 1 Integrative Medicine, Center de Wetering, Leeuwarden; 2 Department of Geriatric Psychiatry and Neuropsychiatry, GGZ-WNB, Halsteren; 3 Integrative Medicine, Leonardo Scientific Research Institute, Groningen, Netherlands

## Abstract

**Introduction:**

Oxidative stress and mitochondrial dysfunction play a central role in the pathophysiology of both depression and cognitive decline. Alpha-lipoic acid (ALA), a naturally occurring antioxidant and mitochondrial cofactor, has been studied for its potential neuroprotective effects. Its ability to cross the blood–brain barrier, regenerate endogenous antioxidants, and improve glucose metabolism makes it a promising adjunctive treatment. This systematic review evaluates the evidence on the efficacy of ALA in depression and cognitive disorders.

**Objectives:**

The aims of this review are to: (a) synthesize available clinical and preclinical evidence on ALA in depression and cognitive impairment, (b) evaluate its therapeutic potential as a stand-alone or adjunctive intervention, and (c) explore proposed biological mechanisms underlying its effects.

**Methods:**

A systematic search was conducted in PubMed, EMBASE, and Cochrane Library for randomized controlled trials (RCTs) and observational studies. Eligible studies assessed the effects of ALA on depressive symptoms or cognitive outcomes, measured by validated clinical scales or neuropsychological tests.

**Results:**

Across identified studies, ALA demonstrated moderate improvements in depressive symptoms, particularly in patients with comorbid metabolic disorders or diabetes. Adjunctive use alongside conventional antidepressants yielded greater benefits than monotherapy. In cognitive disorders, especially Alzheimer’s disease and mild cognitive impairment, ALA slowed cognitive decline and improved memory performance when used alone or in combination with acetyl-L-carnitine. Proposed mechanisms include reduction of oxidative stress, enhancement of mitochondrial function, regulation of neurotransmitters such as dopamine and serotonin, and improvement of insulin sensitivity. However, heterogeneity in study design, small sample sizes, and variable dosing regimens limit the generalizability of findings.

**Conclusions:**

ALA shows potential as an adjunctive treatment for depression and cognitive decline by targeting oxidative stress and metabolic dysfunction. While preliminary results are promising, larger high-quality RCTs are required to confirm efficacy, establish optimal dosage, and clarify long-term safety.

**Disclosure of Interest:**

None Declared

